# Incidence of Dengue Virus Infection in Adults and Children in a Prospective Longitudinal Cohort in the Philippines

**DOI:** 10.1371/journal.pntd.0004337

**Published:** 2016-02-04

**Authors:** Maria Theresa Alera, Anon Srikiatkhachorn, John Mark Velasco, Ilya A. Tac-An, Catherine B. Lago, Hannah E. Clapham, Stefan Fernandez, Jens W. Levy, Butsaya Thaisomboonsuk, Chonticha Klungthong, Louis R. Macareo, Ananda Nisalak, Laura Hermann, Daisy Villa, In-Kyu Yoon

**Affiliations:** 1 Philippines-AFRIMS Virology Research Unit, CAP Building, Cebu City, Philippines; 2 Division of Infectious Diseases and Immunology, Department of Medicine, University of Massachusetts Medical School, Worcester, Massachusetts, United States of America; 3 Cebu City Health Department, Cebu City, Philippines; 4 Department of Epidemiology, Johns Hopkins School of Public Health, Baltimore, Maryland, United States of America; 5 Department of Virology, Armed Forces Research Institute of Medical Sciences, Bangkok, Thailand; 6 Department of Medicine, University of Toronto, Toronto, Ontario, Canada; 7 Dengue Vaccine Initiative, International Vaccine Institute, Seoul, Korea; Oswaldo Cruz Foundation, BRAZIL

## Abstract

**Background:**

The mean age of dengue has been increasing in some but not all countries. We sought to determine the incidence of dengue virus (DENV) infection in adults and children in a prospective cohort study in the Philippines where dengue is hyperendemic.

**Methodology/Principal Findings:**

A prospective cohort of subjects ≥6 months old in Cebu City, Philippines, underwent active community-based surveillance for acute febrile illnesses by weekly contact. Fever history within the prior seven days was evaluated with an acute illness visit followed by 2, 5, and 8-day, and 3-week convalescent visits. Blood was collected at the acute and 3-week visits. Scheduled visits took place at enrolment and 12 months that included blood collections. Acute samples were tested by DENV PCR and acute/convalescent samples by DENV IgM/IgG ELISA to identify symptomatic infections. Enrolment and 12-month samples were tested by DENV hemagglutination inhibition (HAI) assay to identify subclinical infections. Of 1,008 enrolled subjects, 854 completed all study activities at 12 months per-protocol undergoing 868 person-years of surveillance. The incidence of symptomatic and subclinical infections was 1.62 and 7.03 per 100 person-years, respectively. However, in subjects >15 years old, only one symptomatic infection occurred whereas 27 subclinical infections were identified. DENV HAI seroprevalence increased sharply with age with baseline multitypic HAIs associated with fewer symptomatic infections. Using a catalytic model, the historical infection rate among dengue naïve individuals was estimated to be high at 11–22%/year.

**Conclusions/Significance:**

In this hyperendemic area with high seroprevalence of multitypic DENV HAIs in adults, symptomatic dengue rarely occurred in individuals older than 15 years. Our findings demonstrate that dengue is primarily a pediatric disease in areas with high force of infection. However, the average age of dengue could increase if force of infection decreases over time, as is occurring in some hyperendemic countries such as Thailand.

## Author Summary

The average age of dengue has been increasing in some but not all dengue endemic countries. To investigate the age pattern of dengue in people of all ages ≥6 months old, a prospective community-based cohort study was undertaken in Cebu City, Philippines where dengue virus has been circulating for many decades. Active surveillance for acute fevers was performed, and acute/convalescent blood samples were tested for evidence of symptomatic dengue. Blood was also collected at enrolment and one year later, and tested serologically to identify subclinical infections. Overall, 1.62 symptomatic and 7.03 subclinical infections per 100 person-years of surveillance were detected. Among people older than 15 years, only one symptomatic dengue case occurred while 27 subclinical infections were identified. By analyzing age-specific dengue serology data, the historical infection rate among people with no prior dengue virus infection was found to be high at around 11–22% per year. Our results show that dengue is primarily a childhood disease in endemic settings where the historical infection rate has been high. However, the average age of dengue could increase if the infection rate decreases over time as is happening in some endemic countries like Thailand.

## Introduction

Dengue virus (DENV) is the leading cause of vector-borne viral disease globally with an estimated 390 million infections and 96 million symptomatic cases occurring annually [1]. Ecologic and demographic changes are thought to be major contributing factors to the emergence of dengue over the past few decades [2]. DENV infection can present as asymptomatic infection, subclinical infection, undifferentiated fever, dengue fever, dengue hemorrhagic fever (DHF) with or without dengue shock syndrome (DSS), and other severe forms of dengue [3]. Estimates of the proportion of subclinical infections among all DENV infections have ranged widely depending on the year, location, population, and surveillance method [4]. Prospective longitudinal cohort studies can characterize the burden and clinical spectrum of DENV infections including subclinical infections [5].

Multiple exposures to DENV are generally presumed to result in protective immunity leading to lower incidence of clinically overt disease in adults in areas with hyperendemic transmission [6,7]. However, when dengue does occur in adults, the clinical manifestations may be more apparent than in children, perhaps due to differing physiologies and more frequent co-morbid conditions in adults [8–11]. At the same time, some dengue hyperendemic countries have reported an increase in the mean age of dengue [12–15]. An important contributing factor to this age increase may be demographic transition in some countries where decreasing birth and death rates may lead to decreasing force of infection (FOI) due to fewer susceptible individuals entering the population [16,17]. Yet, very few prospective longitudinal cohort studies undergoing active surveillance have been conducted in adults to assess overall incidence and disease burden [18,19]; and even fewer have evaluated dengue incidence and relative proportion of subclinical infections in adults and children within the same cohort.

In the Philippines, a dengue outbreak was first reported as early as 1906 [20], and the first epidemic of severe dengue was documented in Manila in 1953 [21]. Since then, dengue has been hyperendemic in most areas of the country with a general increase in the number of dengue cases over time [22]. We conducted a prospective longitudinal cohort study in Cebu City, Philippines, among subjects of all ages ≥6 months, with the objective of determining the age-stratified incidence of DENV infection and the ratio of symptomatic to subclinical infection in adults and children. We found that in a setting with high force of infection with consequently high DENV seroprevalence in adults, symptomatic infections were rare in individuals older than 15 years with almost all infections in this age group being subclinical.

## Methods

### Ethics Statement

The study was approved by the Institutional Review Boards of Vicente Sotto Memorial Medical Center (VSMMC) in Cebu City, Philippines, and the Walter Reed Army Institute of Research (WRAIR). Written informed consent was obtained from subjects ≥18 years old and from parents of subjects <18 years old. Written assent was obtained from children ≥12 years old.

### Study Location

The study was conducted in the barangay (i.e., village equivalent) of Punta Princesa, an urban community in Cebu City, capital of Cebu province located within the Visayas region of the Philippines. Punta Princesa encompasses 0.96 sq km with a total population of about 27,000 people. Public outpatient care is available to local residents at the Punta Princesa Health Center with patients who require higher level medical care or hospitalization referred to Cebu City Medical Center, the tertiary public hospital of Cebu City, or to VSMMC, the tertiary public hospital of Cebu province.

### Prospective Cohort

Study enrolment and surveillance procedures have been previously described [23]. Subjects were enrolled from March to May 2012 in a prospective longitudinal seroepidemiological cohort study using convenience sampling within the community. Inclusion criteria included age ≥6 months and residence within Punta Princesa. Exclusion criteria included known active pulmonary tuberculosis, in order to decrease risk to study staff. Enrolment was limited to one subject per household. Approximately 1,000 subjects were targeted for enrolment with roughly equal distribution among five different age groups: 6 months-5 years, 6–15 years, 16–30 years, 31–50 years, and >50 years old. At enrolment and at 12 months, subjects were administered demographic and health questionnaires, and underwent blood collections. Blood was processed into serum aliquots within 24 hours of phlebotomy, frozen at approximately -70°C, and eventually shipped on dry ice to the Armed Forces Research Institute of Medical Sciences (AFRIMS) in Bangkok, Thailand for testing. The serum was tested by hemagglutination inhibition (HAI) assay for all four DENV serotypes and Japanese encephalitis virus (JEV).

### Active Surveillance

Enrolled subjects underwent active community-based surveillance to detect acute febrile illness which was defined as reported fever or measured temperature >38.0°C. Subjects were instructed to report any fevers, and were contacted by study staff once a week through telephone, short message service, and/or home visits. A history of fever within the prior seven days triggered an acute fever investigation consisting of an acute illness visit followed by subsequent visits at 2, 5 and 8 days, and a convalescent visit at 3 weeks. Clinical assessments were performed at all visits, and blood was collected at the acute and 3-week convalescent visits. Blood was processed into serum aliquots within 24 hours of phlebotomy, frozen at approximately -70°C and shipped on dry ice to AFRIMS in Bangkok, Thailand. Acute serum was tested by hemi-nested reverse transcriptase polymerase chain reaction (PCR) to detect DENV RNA. Paired acute/convalescent sera were tested by an in-house DENV IgM/IgG capture enzyme-linked immunosorbent assay (ELISA) and by DENV/JEV HAI.

### Laboratory Assays

#### Qualitative DENV RT-PCR/nested PCR

Viral RNA was extracted from 140 μl of serum using viral RNA extraction kit (Qiagen, Valencia, CA, USA). A two-step nested PCR was performed as previously described [24]. The first RT-PCR step used consensus sense and anti-sense primers covering DENV serotypes 1–4. The RT-PCR product was then re-amplified in a second step containing five primers: the original sense primer and four internal, serotype-specific, anti-sense primers.

#### DENV/JEV IgM/IgG capture ELISA

An in-house anti-DENV/JEV IgM/IgG capture ELISA was performed as previously described [25]. Sucrose-acetone extracted viral antigen passaged in suckling mouse brains was utilized. IgM ≥40 units indicated an acute DENV infection. IgM-to-IgG ratio ≥1.8 was considered to be acute primary infection while a ratio <1.8 was considered as acute secondary infection. For paired samples, a two-fold increase in IgG with ≥100 units was considered to be an acute secondary infection even in the absence of IgM ≥40 units.

#### DENV/JEV hemagglutination inhibition assay

HAI serology was performed on acetone-extracted serum using the procedure of Clarke and Casals modified for 96-well V-bottom plates as previously described [26]. Suckling mouse brain passaged, sucrose-extracted viral antigen was added to 25 ul of two-fold serial serum dilutions (1:10 to 1:1280). A ≥four-fold rise in HAI titer for any DENV serotype was considered as a positive seroconversion.

### Study Definitions

Acute symptomatic DENV infection was defined as an acute febrile illness with positive DENV PCR in the acute sample, and/or positive DENV IgM/IgG ELISA in the paired acute/convalescent samples. Subclinical DENV infection was defined as a ≥four-fold rise in DENV HAI titer for any serotype in paired enrolment/12-month samples with no acute symptomatic DENV infection detected during the intervening surveillance period.

Subjects with an HAI titer <10 to all four serotypes were considered to be dengue naive. Those with an HAI titer ≥10 to only one DENV serotype were considered to have monotypic dengue immune status. Those with HAI titers ≥10 to two or more serotypes were considered to have multitypic immune status.

### Statistical Analysis

Descriptive statistics for infection rates, symptoms and other characteristics were performed. To inform the underlying infection dynamics in this population, we employed the chi-square or Fisher’s exact test as appropriate to evaluate the association between DENV infection and both age >15 years and the nominal categorical baseline immune status. To estimate FOI, age-stratified DENV HAI seroprevalence data at enrolment was analyzed such that HAI titer <10 for all four DENV serotypes indicated a dengue naive subject while HAI titer ≥10 for at least one DENV serotype indicated a non-naive subject. A catalytic model [16,27] was used to estimate FOI (λ) for each DENV HAI serotype assuming independence between serotypes (details available in supplementary information). In this catalytic model, (1—exp [-λ]) was the proportion of the dengue naive population infected each year by each serotype. FOI was estimated in a Markov Chain Monte Carlo (MCMC) framework. Means and 95% credible intervals were reported. When using seroprevalence at a single time point, age and time could have been confounded so that changes in FOI during the last five years, for example, could have been due either to differential exposure in individuals <5 years old or to different FOI in the last five years. Two models with different assumptions were assessed; one in which FOI was assumed to be constant over all years (model 1), and one in which FOI could be different during the previous five years (or equivalently, among individuals <5 years old) from the years before then (model 2). For each model, an average FOI was estimated across all serotypes. Ages were rounded down so those <1 year old were shown as 0. All analyses were performed using R version 3.0.2 (R Foundation for Statistical Computing, Vienna, Austria).

## Results

**Fig 1:**
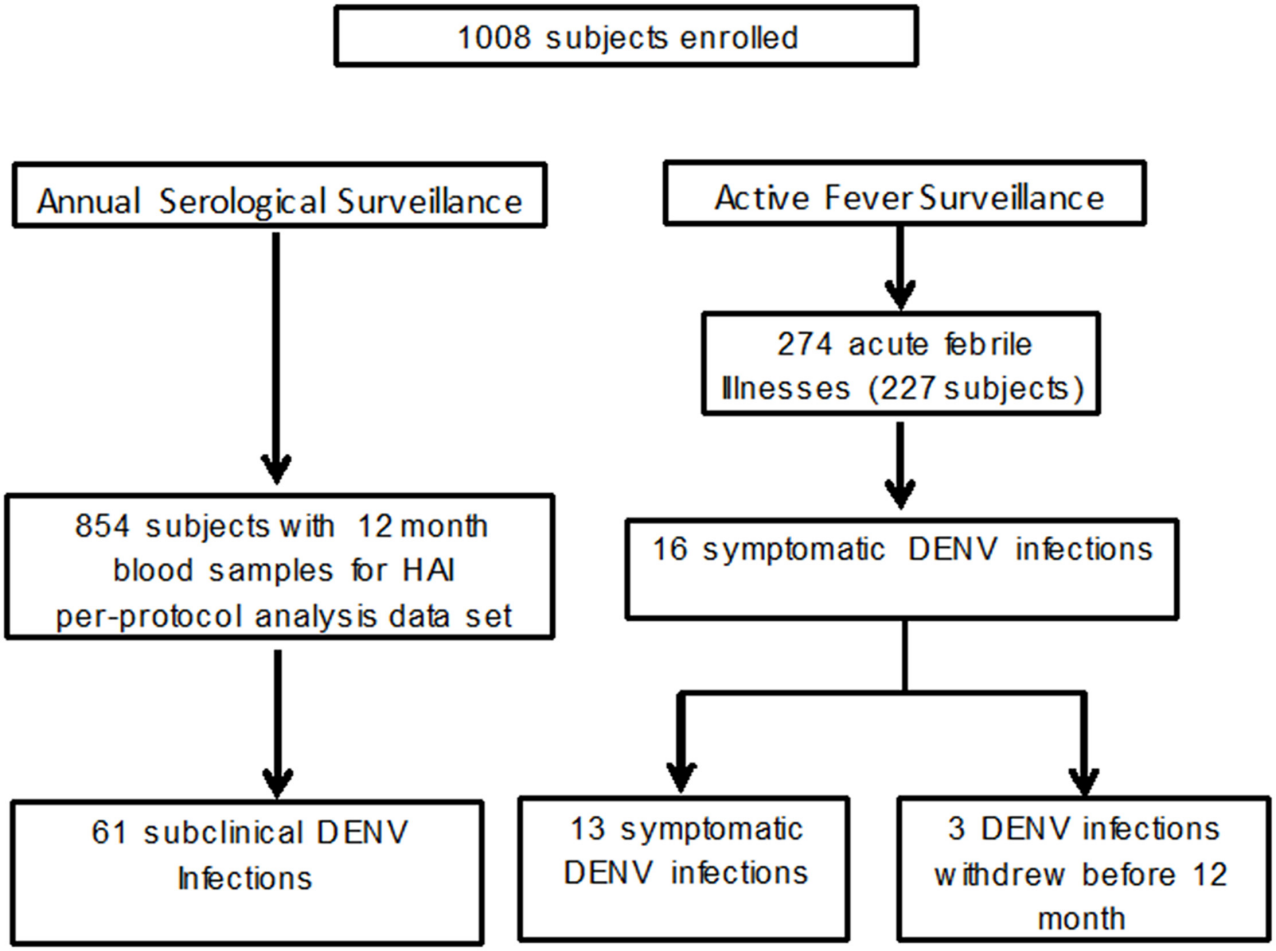
Study flow chart.

A total of 1,008 subjects were enrolled from March to May 2012. A description of the enrolled cohort is presented in Table 1. During 985 person-years of active surveillance from enrolment to 12 months, 274 acute febrile illnesses were detected with 268 acute and 261 convalescent blood samples collected (Fig 1). Of 50,125 weekly contacts for the 1008 enrolled subjects, only 1.2% were unsuccessful due to the subject being “unavailable.” These weekly contacts were completed by household visit (22.4%), SMS (37.6%) or telephone call (38.8%). Sixteen acute symptomatic DENV infections were identified during this surveillance period. Thirteen of the 16 symptomatic infections were PCR-positive with serotype data available: 10 DENV-1, two DENV-2 and one DENV-3. A total of 854 subjects completed all study activities by the 12-month visit constituting the “per-protocol” subjects. Of the 154 subjects who did not complete all study activities, 66 relocated out of the study area, 64 withdrew consent, 16 were lost to follow up, and eight developed other physical conditions or problems. Subjects in the youngest age group had the largest number who did not complete all study activities (Table 1). Subclinical infections were only possible for per-protocol subjects since only this group had DENV HAI testing performed at 12 months. During 868 person-years of active surveillance in just the per-protocol subjects, 13 acute symptomatic DENV infections and 61 subclinical infections occurred.

**Table 1:** Description of cohort subjects.

Characteristic	Enrolled, n (%)	Per-protocol, n (%)*
Total subjects	1008 (100)	854 (100)
Age group		
6 mos—5 yrs	203 (20.1)	148 (17.3)
6–15 yrs	201 (19.9)	184 (21.5)
16–30 yrs	200 (19.8)	168 (19.7)
31–50 yrs	204 (20.2)	172 (20.1)
>50 yrs	200(19.8)	182 (21.3)
Male gender	500 (49.6)	415 (48.6)
Number in household		
1	16 (1.6)	15 (1.8)
2–3	207 (20.5)	174 (20.4)
4–6	526 (52.2)	439 (51.4)
7–10	237 (23.5)	205 (24.0)
>10	22 (2.2)	21 (2.5)
Number of children in household		
0	199 (19.7)	172 (20.1)
1	231 (22.9)	190 (22.2)
2	229 (22.7)	188 (22.0)
3	180 (17.9)	156 (18.3)
>3	169 (16.8)	148 (17.3)

*Per-protocol subjects completed all study activities at 12 months including enrollment and 12-month blood collections.

n = number.

The incidence of symptomatic DENV infection per 100 person-years in the overall cohort (including non-per-protocol subjects) was 1.62 (95%CI: 0.97, 2.58). Only one symptomatic infection occurred in subjects >15 years of age. The incidence of subclinical infection per 100 person-years in per-protocol subjects was 7.03 (95%CI: 5.42, 8.96), ranging from 4.16 in the 31–50 year group, to 10.04 in the 6–15 year group (Table 2). Three subjects were hospitalized for DHF, and five subjects received outpatient medical care (S1 Table), and no fatalities occurred.

**Table 2:** Incidence of symptomatic and subclinical dengue virus (DENV) infections in different age groups.

Age	Subjects, n^a^	Symptomatic DENV infection, n [n/100 person-yrs(95% CI)]^b^	Subclinical DENV infection, n [n/100 person-yrs(95% CI)]^a^	Total DENV infection, n/100 person-yrs	Ratio of subclinical to symptomatic DENV infection
6 mos—5 yrs	148	5 [2.50 (0.95, 5.49)]	15 [9.69 (5.66, 15.59)]	12.19	3.9:1
6–15 yrs	184	10 [4.90 (2.52, 8.70)]	19 [10.04 (6.25, 15.35)]	14.94	2.0:1
16–30 yrs	168	1 [0.5 (0.04, 2.31)]	12 [6.79 (3.71, 11.50)]	7.29	13.6:1
31–50 yrs	172	0 [0 (0, 1.32)]	7 [4.16 (1.85, 8.17)]	4.16	^c^
>50 yrs	182	0 [0 (0, 1.29)]	8 [4.46 (2.11, 8.42)]	4.46	^d^
All ages	854	16 [1.62 (0.97, 2.58)]	61 [7.03 (5.42, 8.96)]	8.65	4.3:1

^a^Based on 854 per-protocol subjects with both enrolment and 12-month blood collections.

^b^Based on all subjects (per-protocol and non per-protocol)

^c^Seven subclinical DENV infections and no symptomatic infections.

^d^Eight subclinical DENV infections and no symptomatic infections.

DENV = dengue virus; n = number; CI = confidence interval.

**Fig 2:**
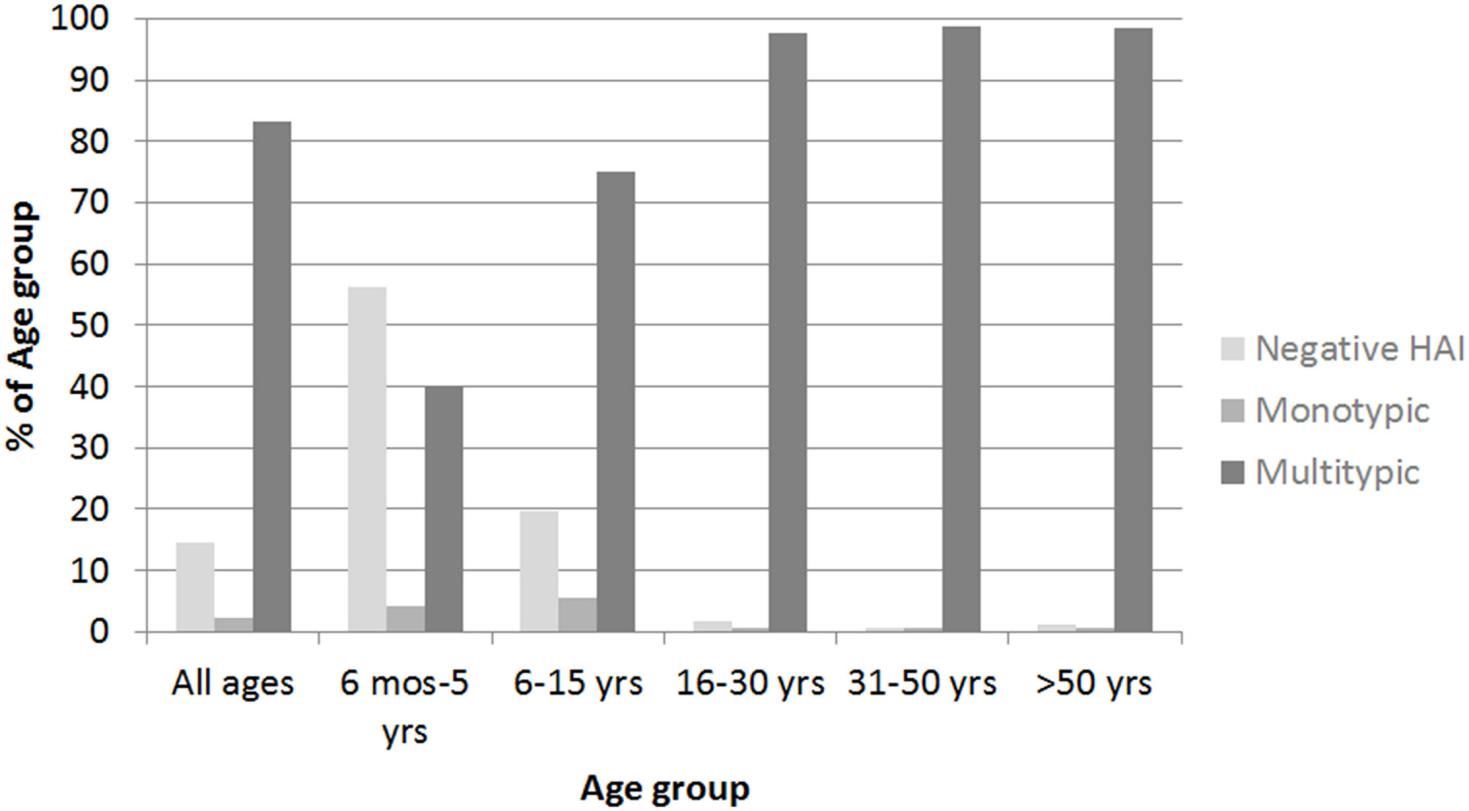
Dengue virus (DENV) hemagglutination inhibition (HAI) profiles at enrolment in different age groups.

DENV HAI seroprevalence increased sharply with age (Fig 2). The proportion of each age group that had a negative HAI profile at enrolment decreased from 56.1% in 6 month-5 year olds to 0.6% in 31.50 year olds, while the proportion having multitypic HAIs increased from 39.9% in 6 month-5 year olds to 98.8% in 31–50 year olds. The seroprevalence of multitypic HAIs was >98.3% for all ages >15 years.

**Fig 3:**
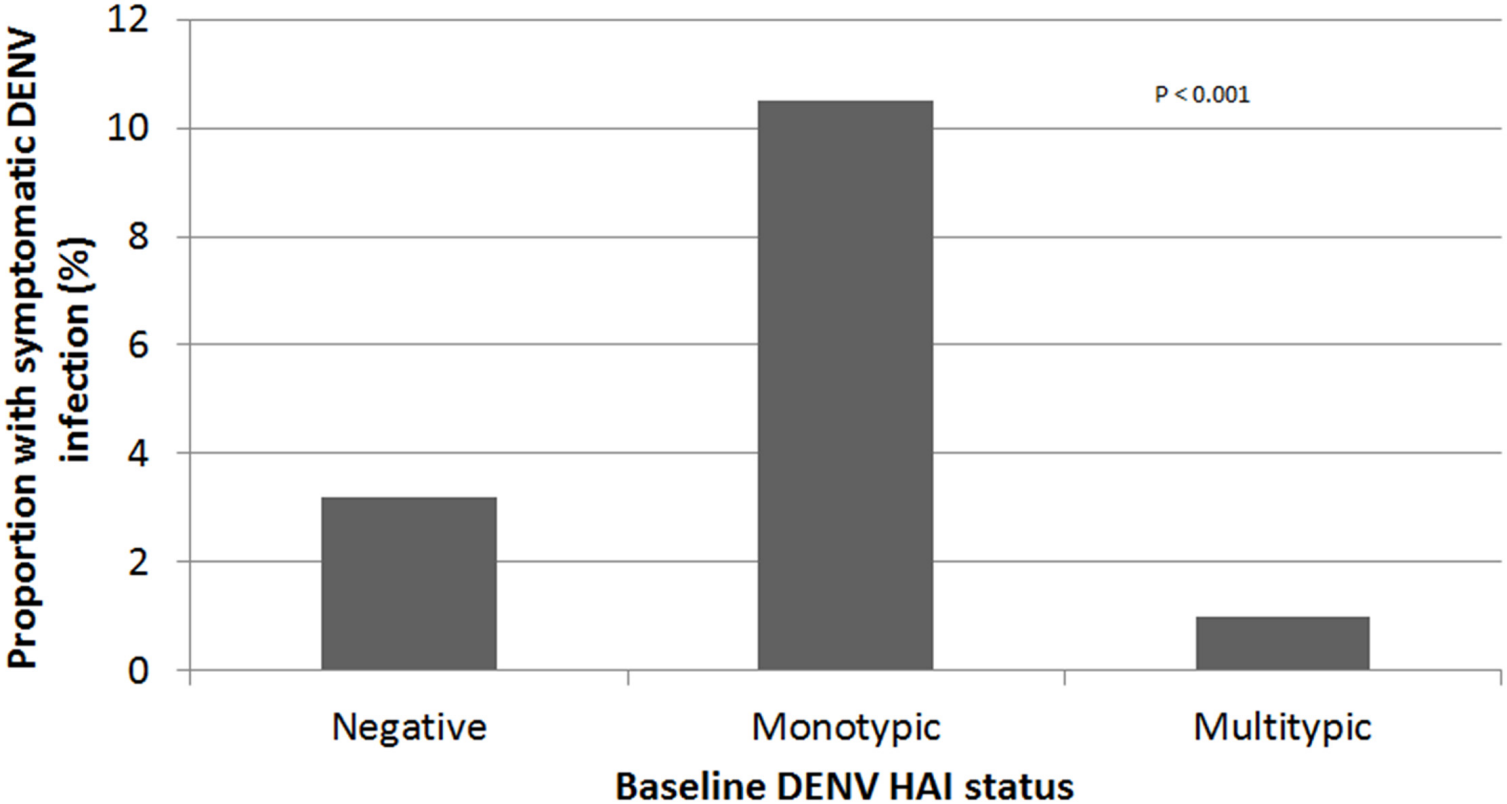
Proportion of symptomatic dengue virus (DENV) infections among all per-protocol subjects with different DENV hemagglutination inhibition (HAI) profiles at enrolment. Negative HAI: 4/125; monotypic HAI: 2/19; multitypic HAI: 7/710 (chi-square, p<0.001). Three symptomatic cases in non-per-protocol subjects were not included because HAI testing was not completed.

**Fig 4:**
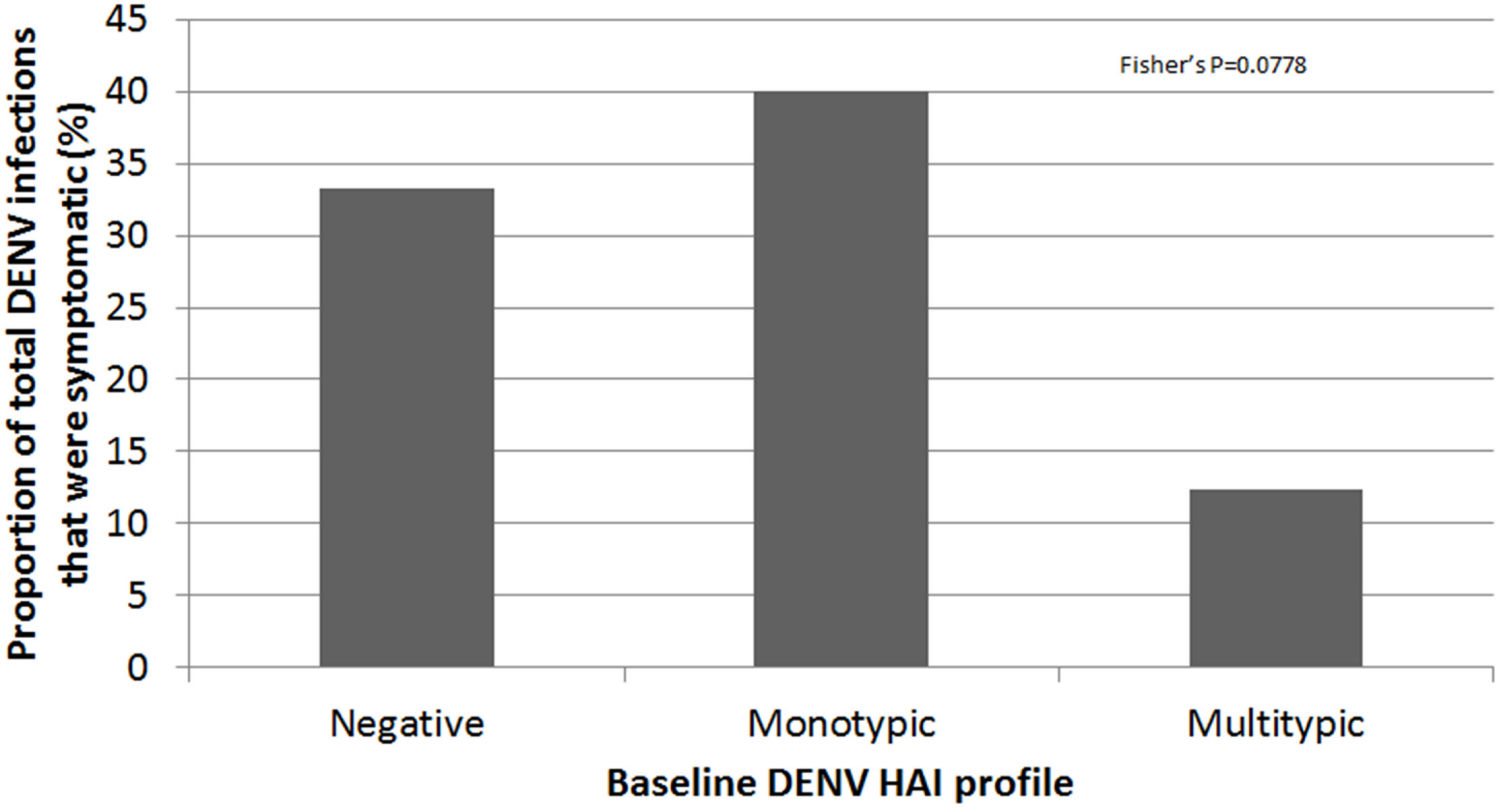
Proportion of symptomatic dengue virus (DENV) infections among total (symptomatic and subclinical) DENV infections in per-protocol subjects with different DENV hemagglutination inhibition (HAI) profiles at enrolment. Negative HAI: 4/12; monotypic HAI: 2/5; multitypic HAI: 7/57 (chi-square, p = 0.068). Three symptomatic cases were not included because of missing HAI titers.

The proportion of subjects who developed symptomatic DENV infection differed depending on baseline DENV HAI profile (Fig 3). Symptomatic infections occurred more often with monotypic (2 of 19) and negative (4 of 125) HAIs at enrolment, and less often with multitypic (7 of 710) HAIs (chi-square, p<0.001). In addition, the proportion of symptomatic infections among all DENV infections (i.e., symptomatic and subclinical) tended to differ depending on the HAI profile at enrolment (chi-square, p = 0.086) (Fig 4).

**Fig 5:**
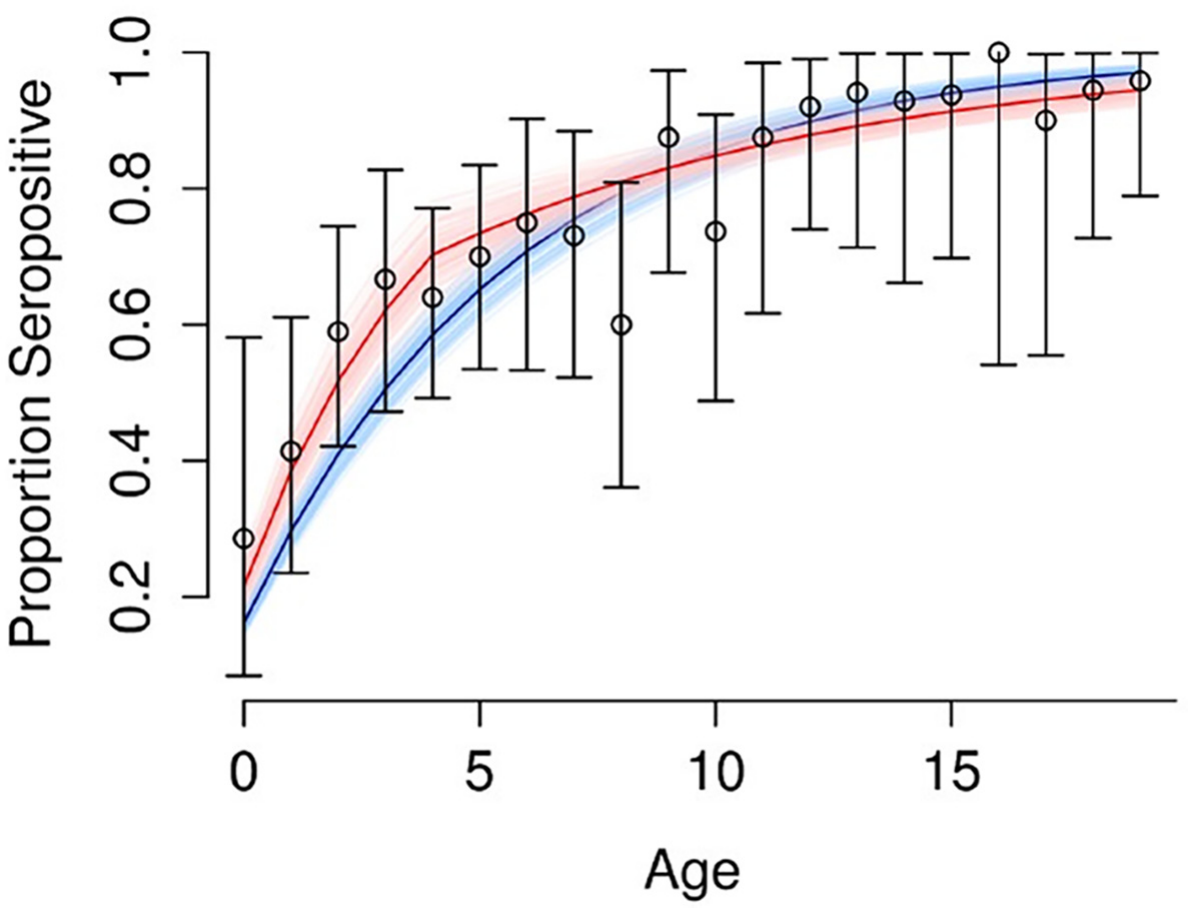
Proportion of subjects of different ages that were dengue seropositive at enrolment (shown in black). Fit of model 1 assumed constant force of infection (shown in blue). Fit of model 2 assumed non-constant force of infection (shown in red).

**Fig 6:**
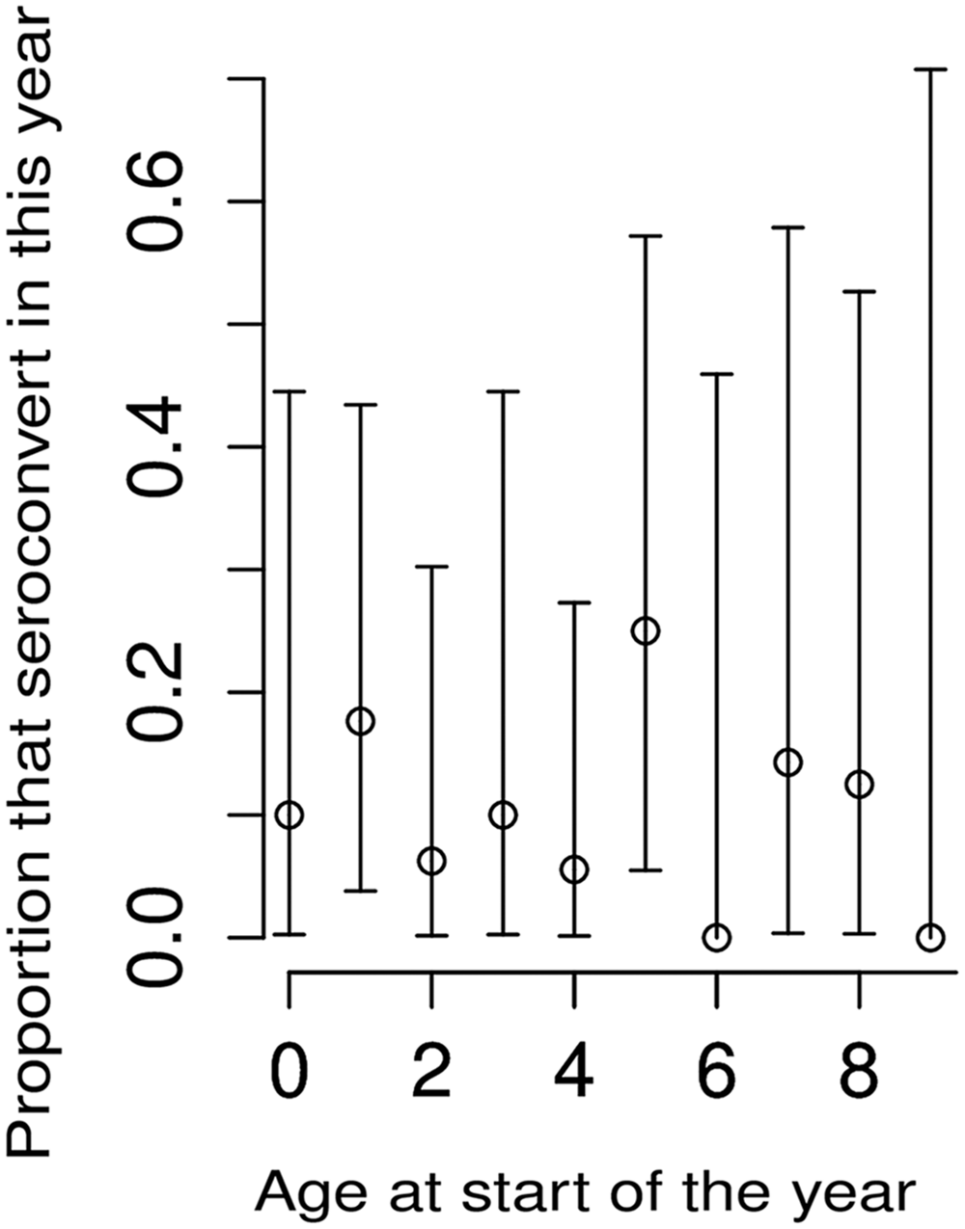
Proportion of dengue naïve subjects at enrolment that seroconverted at 12 months.

FOI was estimated by analyzing age-stratified DENV HAI seroprevalence at enrolment. Model 1, which assumed a constant FOI, yielded an average FOI of 0.044/year (95% CI: 0.039, 0.049) for each HAI serotype over the previous 20 years, with a log likelihood of -272 (nearly universal HAI seropositivity in adults older than 20 years did not allow FOI estimates beyond the previous 20 years). The model fit is shown in dark blue in Fig 5 with uncertainty shown in light blue. Model 2, which assumed non-constant FOI, yielded an average FOI of 0.061/year per serotype (95% CI: 0.051, 0.072) during the previous five years and 0.028/year per serotype (95% CI: 0.020, 0.036) for the 15 years before then, with a log likelihood of -265. The model fit is shown in red in Fig 5, with uncertainty shown in pink. Model 2 fit the data slightly better, particularly in the younger age groups, suggesting either that there was a higher FOI in the previous five years, or that subjects ≤5 years of age experienced a higher FOI than older subjects. To assess whether different age groups may have had differing FOIs, DENV HAI seroconversions at 12 months in subjects who were dengue naive at enrolment were analyzed by age group (Fig 6). The age given in Fig 6 is the age at the start of the year in which seroconversions were considered. Because the number of dengue naive subjects was small (n = 125), there were large confidence intervals for these proportions. Nevertheless, there was no evidence of differential seroconversion in subjects ≤5 years old than those >5 years, suggesting that the observed results from model 2 were due to FOI being higher during the previous five years than before then, rather than to differential exposure with age.

## Discussion

In our study in a hyperendemic area of the Philippines, symptomatic DENV infections rarely occurred in subjects older than 15 years. Infections that did occur in this older age group were more likely to be subclinical than in those ≤15 years. This age pattern of DENV infection occurred in the setting of high seroprevalence of multitypic DENV HAIs in adults likely due to high force of infection. This is one of the first prospective longitudinal cohort studies using active surveillance in which dengue incidence in adults and children have been determined within the same cohort.

The historical force of infection based on age-stratified seroprevalence at enrolment was found to be high in our study. Presuming a constant FOI, the infection rate among dengue naïve individuals was approximately 16% per year over the previous 20 years (based on FOI of 0.044/year per serotype). Presuming a non-constant FOI, the infection rate was about 22% per year during the previous five years (FOI of 0.061/year per serotype) and 11% per year before then (FOI of 0.028/year per serotype). Whichever of these two models was used, the results indicate that sustained DENV transmission has been ongoing in Cebu for many years. By the time subjects reached 16 years of age, over 96% had multitypic DENV HAIs. The high population turnover in the Philippines leading to large numbers of dengue naïve individuals entering the population may be a contributing factor to the high FOI. In 2014, the birth and death rates in the Philippines were 25.34 and 5.02 per 1,000 population, respectively, as compared to 12.95 and 7.29 in Thailand (World Health Statistics 2014, World Health Organization). Nevertheless, other factors may also be playing a role in the Philippines, such as permissive environmental/ecological factors and/or incomplete vector control. The predominant viral serotype and strain during different years may have also affected the epidemic potential and, thus, FOI. The current epidemiology of dengue in the Philippines may mirror past epidemiology in other countries such as Thailand before demographic transition occurred.

We were unable to perform a sub-analysis of adult symptomatic dengue because so few symptomatic infections in adults occurred. This lack of cases existed in the setting of high seroprevalence of multitypic HAIs in adults. So while the independent effects of age and HAI profile on symptomatic dengue could not be evaluated, the lack of symptomatic infections in adults was likely related to the high seroprevalence of multitypic HAIs, which in turn was probably due to high historical FOI. It is notable that so few symptomatic infections occurred in subjects >15 years old despite a vigorous active surveillance system. The incidence of symptomatic dengue in children in our study was comparable to previous prospective cohort studies conducted in children in which the average rate has ranged from 0.6% to 3.9% per year [28], supporting the validity of our surveillance procedures for detecting dengue cases. Our findings demonstrate the primarily pediatric focus of dengue when FOI is high, and why dengue has been considered a childhood disease in many hyperendemic countries. Our study does not, however, clarify whether the clinical manifestations of dengue are more or less severe in adults since so few symptomatic adult cases were observed.

Symptomatic DENV infections occurred more frequently with baseline negative or monotypic HAIs than with multitypic HAIs. A multitypic HAI profile can result from two or more past DENV infections by different serotypes, although a single infection is still possible. If a monotypic HAI profile was presumed to have resulted from a single past infection, then our findings suggest that a second infection with a different serotype may be more likely to cause symptomatic infection.

There were several limitations to our study. First, only one year of surveillance data was available. Given the known cyclical nature of DENV transmission, other years may have different epidemiological and clinical patterns. For example, different predominating DENV serotypes or strains in the context of different population immunity in different years may lead to different ratios of symptomatic and subclinical disease [29]. Second, relatively few symptomatic infections occurred in the overall cohort limiting the statistical significance of our analyses. Third, the use of DENV HAI to determine both seroprevalence and seroconversion may have underestimated the actual rates given the unclear longevity of HAI titers. Measurement of more persistent markers of past infection would have been preferable, but were not performed due to resource limitations. It is, therefore, possible that the incidence of total DENV infections was actually higher than indicated by our study results. Nevertheless, the lack of symptomatic infections observed in adults would not have been affected.

In summary, our study demonstrates that symptomatic dengue is primarily a pediatric disease in hyperendemic areas with high force of infection. However, the average age of dengue could increase if force of infection decreases over time, which may be occurring in some dengue hyperendemic countries such as Thailand.

## Supporting Information

S1 Table Number of completed acute illness visits and follow up visits 1 after detection of acute febrile episodes (n = 274). (TIF)

S2 Table Clinical presentation of symptomatic dengue virus (DENV) infections. (TIF)

S1 Checklist STROBE checklist. (PDF)

S1 Dataset Aggregated data. (XLSX)
