# Supplementary figures and images for: Incidence of Dengue Virus Infection in Adults and Children in a Prospective Longitudinal Cohort in the Philippines
Source: PLoS Negl Trop Dis. 2016 Feb 4;10(2):e0004337. doi: 10.1371/journal.pntd.0004337 (PMC4742283; doi:10.1371/journal.pntd.0004337)

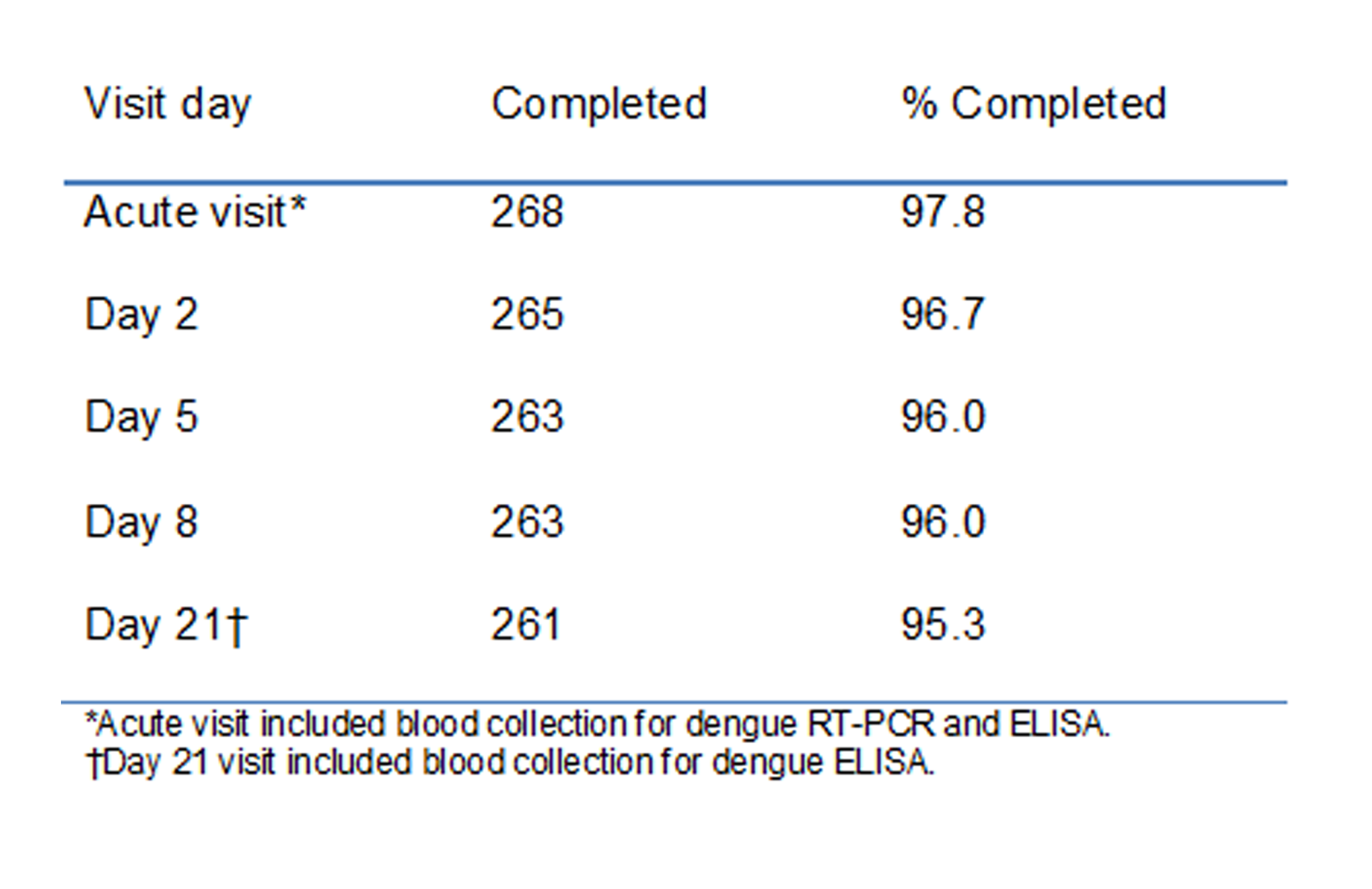

Supplement: S1 Table — (TIF) [file pntd.0004337.s001.tif]

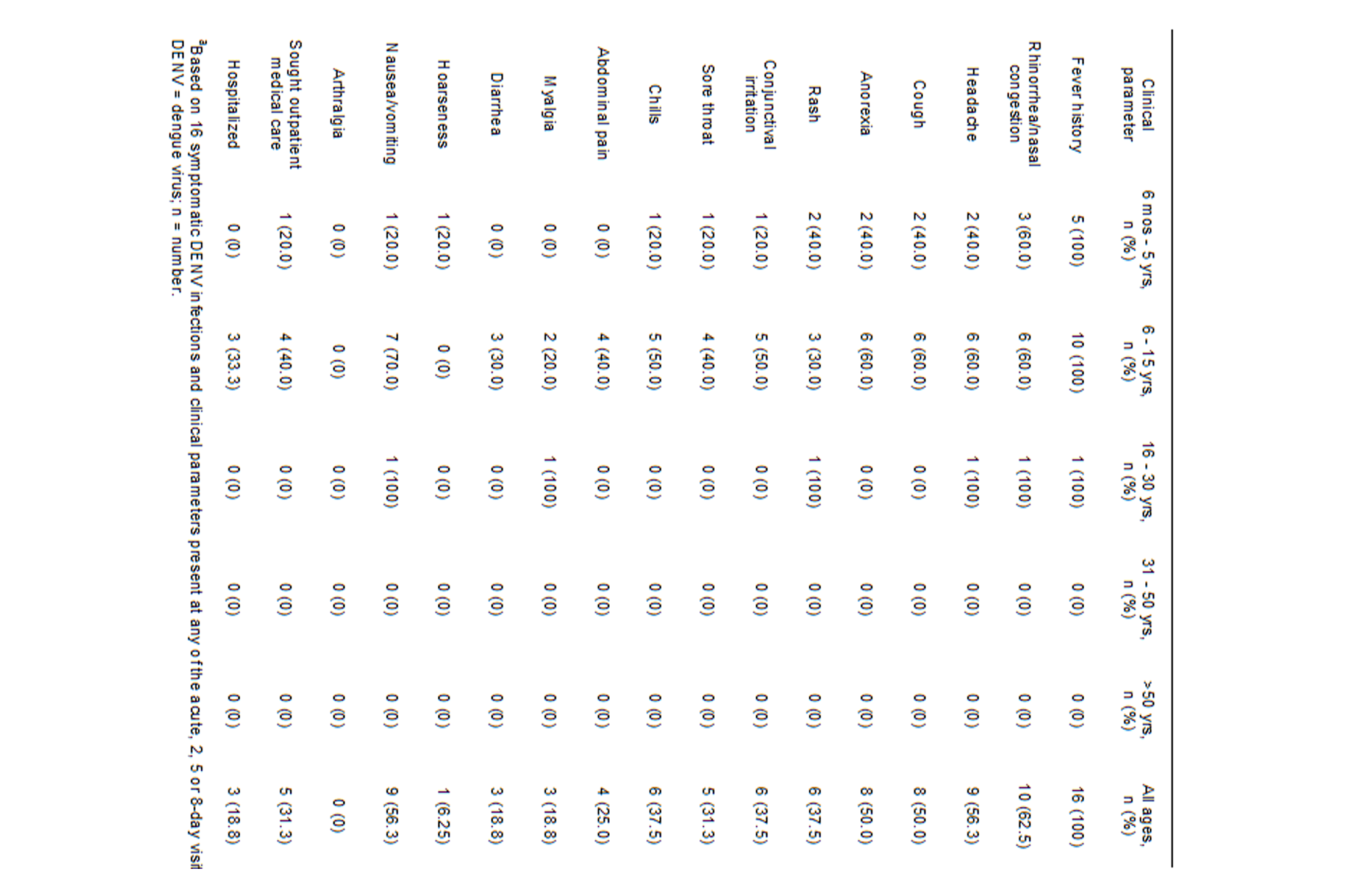

Supplement: S2 Table — (TIF) [file pntd.0004337.s002.tif]
