# Supplementary material for: Incidence of Dengue Virus Infection in Adults and Children in a Prospective Longitudinal Cohort in the Philippines
Source: PLoS Negl Trop Dis. 2016 Feb 4;10(2):e0004337. doi: 10.1371/journal.pntd.0004337 (PMC4742283; doi:10.1371/journal.pntd.0004337)
Supplement: S1 Checklist — (PDF) [file pntd.0004337.s003.pdf]

STROBE Statement—Checklist of items that should be included in reports of ***cohort studies***

	Item No	Recommendation
**Title and abstract**	1	(*a*) Indicate the study's design with a commonly used term in the title or the abstract<br>**The title states "prospective cohort."**
		(*b*) Provide in the abstract an informative and balanced summary of what was done and what was found<br>**This was provided in the abstract.**
**Introduction**		
Background/rationale	2	Explain the scientific background and rationale for the investigation being reported<br>**The introduction states: "Yet, very few prospective longitudinal cohort studies undergoing active surveillance have been conducted in adults to assess overall incidence and disease burden [18,19]; and even fewer have evaluated dengue incidence and relative proportion of subclinical infections in adults and children within the same cohort."**
Objectives	3	State specific objectives, including any prespecified hypotheses<br>**The introduction states: "In order to elucidate the incidence of symptomatic and subclinical DENV infections in adults as well as children, we conducted a prospective longitudinal cohort study in Cebu City, Philippines, among subjects of all ages ≥6 months."**
**Methods**		
Study design	4	Present key elements of study design early in the paper<br>**This was presented early in the Methods section under the sub-section "Prospective Cohort."**
Setting	5	Describe the setting, locations, and relevant dates, including periods of recruitment, exposure, follow-up, and data collection<br>**This was described in the Methods section under the sub-sections "Study Location" and "Prospective Cohort."**
Participants	6	(*a*) Give the eligibility criteria, and the sources and methods of selection of participants. Describe methods of follow-up.<br>**This was described in the Methods section under the sub-sections "Prospective Cohort" and "Active Surveillance."**
		(*b*) For matched studies, give matching criteria and number of exposed and unexposed<br>**Not applicable.**
Variables	7	Clearly define all outcomes, exposures, predictors, potential confounders, and effect modifiers. Give diagnostic criteria, if applicable<br>**This was described in the Methods section under the sub-section "Prospective Cohort." Laboratory definitions were described in the Methods section under the sub-section "Laboratory Assays." Symptomatic DENV infections were defined as illnesses with reported history of fever with specific laboratory criteria. Subclinical infections were defined as those without reported history of fever but with certain laboratory criteria.**
Data sources/ measurement	8*	For each variable of interest, give sources of data and details of methods of assessment (measurement). Describe comparability of assessment methods if there is more than one group<br>**This was described in the Methods section under the sub-section "Prospective Cohort" and "Active Surveillance." Laboratory definitions were described in the Methods section under the sub-section "Laboratory Assays."**
Bias	9	Describe any efforts to address potential sources of bias

		**This was described in the Methods section under the sub-section "Prospective Cohort" and "Active Surveillance." A roughly equal number of subjects were targeted for recruitment from each age group. Only one subject was recruited from each household.**
Study size	10	Explain how the study size was arrived at<br>**Since this was an exploratory study, the target size of the cohort was largely based on logistical considerations.**
Quantitative variables	11	Explain how quantitative variables were handled in the analyses. If applicable, describe which groupings were chosen and why<br>**Age groups for enrolment were determined prior to study initiation based on commonly used age categories. Since this was an exploratory study, the age groupings were arbitrary. Laboratory definitions were described in the Methods section under the sub-section "Laboratory Assays." Definitions of symptomatic and subclinical DENV infections are described in the Methods section under the sub-section "Study Definitions."**
Statistical methods	12	(*a*) Describe all statistical methods, including those used to control for confounding<br>**These are described in the Methods section under the sub-section "Statistical Analysis" and also in the supplemental file describing force of infection calculations.**
		(*b*) Describe any methods used to examine subgroups and interactions<br>**These are described in the Methods section under the sub-section "Statistical Analysis" and also in the supplemental file describing force of infection calculations. The "Discussion" also provides an explanation that "We were unable to perform a sub-analysis of adult symptomatic dengue because so few symptomatic infections in adults occurred."**
		(*c*) Explain how missing data were addressed<br>**Any relevant missing data were listed in the paper. These were minimal and were not included in any analyses.**
		(*d*) If applicable, explain how loss to follow-up was addressed<br>**Subjects who completed all protocol activities were designated as "per-protocol" subjects. Most of the analyses such as determination of subclinical infection rates and calculation of force of infection were only able to be performed on "per protocol" subjects who completed all study activities.**
		(*e*) Describe any sensitivity analyses<br>**Not performed.**
**Results**		
Participants	13*	(a) Report numbers of individuals at each stage of study—eg numbers potentially eligible, examined for eligibility, confirmed eligible, included in the study, completing follow-up, and analysed<br>**These were listed in the Results section and in Figure 1.**
		(b) Give reasons for non-participation at each stage<br>**Most of the analyses could only be done on "per-protocol" subjects who completed all study activities. However, compliance with the protocol procedures was generally very good.**
		(c) Consider use of a flow diagram<br>**See Figure 1.**
Descriptive data	14*	(a) Give characteristics of study participants (eg demographic, clinical, social) and information on exposures and potential confounders<br>**See Table 1.**

		(b) Indicate number of participants with missing data for each variable of interest<br>**There was no relevant missing data. Most of the basic analyses could only be done on "per-protocol" subjects who completed all study activities.**
		(c) Summarise follow-up time (eg, average and total amount)<br>**This is summarized throughout the paper as person-years of surveillance. In addition, much of the analyses could only be performed on "per-protocol" subjects.**
Outcome data	15*	Report numbers of outcome events or summary measures over time<br>**This is reported in the Results section.**
Main results	16	(*a*) Give unadjusted estimates and, if applicable, confounder-adjusted estimates and their precision (eg, 95% confidence interval). Make clear which confounders were adjusted for and why they were included<br>**This is provided in the Results section.**
		(*b*) Report category boundaries when continuous variables were categorized<br>**Hemagglutination inhibition titers were categorized as negative (<10) or positive (≥10).**
		(*c*) If relevant, consider translating estimates of relative risk into absolute risk for a meaningful time period<br>**Not applicable.**
Other analyses	17	Report other analyses done—eg analyses of subgroups and interactions, and sensitivity analyses<br>**Relevant further analyses are presented in the Results section.**
**Discussion**		
Key results	18	Summarise key results with reference to study objectives<br>**Symptomatic and subclinical DENV infection rates are summarized in the Discussion section.**
Limitations	19	Discuss limitations of the study, taking into account sources of potential bias or imprecision. Discuss both direction and magnitude of any potential bias<br>**Relevant study limitations are described in a separate paragraph in the Discussion section.**
Interpretation	20	Give a cautious overall interpretation of results considering objectives, limitations, multiplicity of analyses, results from similar studies, and other relevant evidence<br>**This is provided in the Discussion section.**
Generalisability	21	Discuss the generalisability (external validity) of the study results<br>**This is mentioned in the Discussion section.**
**Other information**		
Funding	22	Give the source of funding and the role of the funders for the present study and, if applicable, for the original study on which the present article is based<br>**The funding sources were mentioned.**

*Give information separately for exposed and unexposed groups.

Note: An Explanation and Elaboration article discusses each checklist item and gives methodological background and published examples of transparent reporting. The STROBE checklist is best used in conjunction with this article (freely available on the Web sites of PLoS Medicine at http://www.plosmedicine.org/, Annals of Internal Medicine at http://www.annals.org/, and Epidemiology at http://www.epidem.com/). Information on the STROBE Initiative is available at http://www.strobe-statement.org.
